# Drug‐Related Side Effects and Contributing Risk Factors in Children With Congenital Heart Disease: A Cross‐Sectional Study

**DOI:** 10.1002/hsr2.70835

**Published:** 2025-05-19

**Authors:** Esmaeel Toni, Haleh Ayatollahi, Reza Abbaszadeh, Alireza Fotuhi Siahpirani

**Affiliations:** ^1^ Student Research Committee Iran University of Medical Sciences Tehran Iran; ^2^ Health Management and Economics Research Center, Health Management Research Institute Iran University of Medical Sciences Tehran Iran; ^3^ Heart Research Center, Rajaie Cardiovascular Medical and Research Center Iran University of Medical Sciences Tehran Iran; ^4^ Institute of Biochemistry and Biophysics (IBB), Department of Bioinformatics University of Tehran Tehran Iran

**Keywords:** children, congenital heart disease, drug‐related side effects, risk factors

## Abstract

**Background and Aims:**

Children with congenital heart disease (CHD) often require complex pharmacotherapy for symptom management and complication prevention. However, their unique physiological profiles increase vulnerability to drug‐related side effects. This study aimed to identify specialists' perspectives on drug‐related side effects and associated risk factors in pediatric CHD patients.

**Methods:**

A cross‐sectional study was conducted in 2024 involving 20 pediatric cardiologists and pediatric cardiology fellows. Data were collected using two 5‐point Likert scale questionnaires assessing commonly prescribed drugs, observed side effects, and associated risk factors in pediatric CHD patients. Data were analyzed using student's *t*‐tests and descriptive statistics.

**Results:**

According to the findings, the most frequent side effects linked to common medications were hypokalemia (Furosemide; 4.5 ± 0.69), apnea (Prostaglandin E1; 4.5 ± 0.62), and bradycardia (Sotalol; 4.41 ± 0.51). Dosage and polypharmacy emerged as major risk factors, particularly for drugs like Digoxin and Heparin. Younger age, underlying health conditions, and specific drug combinations also increased the risk of side effects. The *t*‐test revealed significant associations between participants' demographics (sex, age, and work experience) and their perceptions of drug‐related side effects and risk factors.

**Conclusions:**

The findings emphasize the need for a personalized approach to pharmacotherapy in pediatric CHD patients, requiring careful drug selection, dose optimization, and enhanced monitoring strategies. Drug‐related side effects highlight the importance of implementing clinical decision support systems, routine therapeutic drug monitoring, and individualized dosing adjustments to mitigate risks. Future research should prioritize longitudinal studies to establish causality relationships, optimize treatment protocols, and improve medication safety in this vulnerable population.

## 1 Introduction

Congenital heart disease (CHD) refers to a range of structural and functional heart defects present at birth and can affect the normal blood flow and potentially impact the pulmonary, systemic, or coronary circulations [1]. The severity of these malformations varies widely—from minor asymptomatic conditions to critical defects requiring immediate intervention [1, 2]. CHD remains one of the most common congenital anomalies globally and is a major contributor to pediatric morbidity and mortality. The global prevalence of CHD is estimated at approximately 9 per 1000 live births, although rates vary by region and population [3, 4]. In Iran, recent studies reported a comparable prevalence of 8–10 per 1000 live births, underscoring the persistent burden of this condition on healthcare systems [4, 5, 6]. Due to advances in medical and surgical interventions, many children with CHD now survive into adolescence and adulthood; however, they continue to face substantial risks of complications, frequent hospitalizations, and reduced quality of life [5, 6].

Pharmacotherapy is essential in the lifelong management of pediatric patients with CHD, serving to alleviate symptoms, reduce the risk of complications, and support their unique physiological needs [7]. Commonly prescribed medications for children with CHD include diuretics, inotropes, and anticoagulants [8]. For many children, long‐term pharmacotherapy is necessary to maintain cardiac function, prevent heart failure, and mitigate thromboembolism and arrhythmias risks [9]. However, due to immature organ systems, pediatric patients often metabolize drugs differently than adults, potentially leading to unpredictable side effects [10]. Specifically, the immature liver and renal functions in children can alter drug metabolism and excretion, sometimes resulting in an increase in susceptibility to drug‐related side effects [11]. Moreover, ethical limitations in conducting experimental studies in pediatric populations further constrain the ability to conduct rigorous drug safety trials, contributing to significant evidence gaps regarding the safety and efficacy of many commonly prescribed medications in this vulnerable group [12].

Despite the increasing prevalence of CHD among pediatric and young adult populations, there is a lack of comprehensive knowledge regarding medication side effects and safety profiles specific to these patients [8, 13]. Current knowledge largely relies on extrapolations from adult studies or limited pediatric trials, which may not adequately reflect the unique risk profiles in children with CHD [13]. This evidence gap poses a critical challenge for clinicians, who must often make medical decisions with limited information on the medication‐specific side effects in paediatric patients. Therefore, the objective of the current study was to identify specialists' perspectives on drug‐related side effects and their associated risk factors in pediatric patients with CHD.

## 2 Methods

### 2.1 Study Design and Setting

This cross‐sectional study was conducted in 2025, and aimed to identify the most commonly prescribed medications, their associated side effects, and risk factors contributing to drug‐related side effects in pediatric patients with CHD. The study followed the Strengthening the Reporting of Observational Studies in Epidemiology (STROBE) guidelines to ensure methodological rigor, transparency, and completeness in reporting [14]. The research settings were three teaching hospitals affiliated with a medical university.

### 2.2 Participants

A total of 32 eligible pediatric cardiologists and pediatric cardiology fellows were invited to participate in the study, of whom only 20 completed the questionnaires.

### 2.3 Data Collection Tools and Procedures

Two 5‐point Likert scale questionnaires (ranging from 1 = Strongly Disagree to 5 = Strongly Agree) were developed based on previous scoping review studies [13, 15]. The first questionnaire had two main sections. The first section collected the participants' demographic data such as age, sex, and years of clinical experience. In the second section, the most common medications prescribed for children with CHD and their associated side effects were listed. In addition, an open‐ended question was considered for each part to allow the respondents to list any other side effects they might have observed previously. The second questionnaire investigated specific risk factors, such as dosage, age, weight, gender, polypharmacy, and underlying conditions for drug‐related side effects in children with CHD. Participants were first asked whether they prescribed each medication (Yes/No). Those who responded “Yes” were then prompted to identify associated side effects and risk factors for that specific medication.

### 2.4 Validity and Reliability

Validity and reliability of the questionnaires were examined to ensure robust data collection. Face validity was assessed by three senior pediatric cardiologists, who evaluated each item for clinical relevance and clarity. Based on their feedback, minor revisions were made to enhance alignment with clinical language and practices, ensuring the questionnaires accurately reflected current standards.

Reliability was examined using the test–retest method over a 2‐week interval with 12 pediatric cardiologists. Intraclass correlation coefficients (ICC) indicated high reliability, with scores of 0.82 for the first and 0.79 for the second questionnaire. These results confirmed a satisfactory test–retest reliability result (*r* = 0.80).

### 2.5 Data Collection Process

Questionnaires were distributed both in person and electronically via email to maximize participant engagement. Personalized invitations were sent to all eligible participants, and follow‐up reminder emails were issued to non‐respondents 1 week later. The data collection period spanned 4 weeks, allowing ample time for responses and follow‐up communications.

### 2.6 Statistical Analysis

Descriptive statistics, including frequency, percentage, mean value, and standard deviations, were calculated to summarize the collected data. The level of agreement regarding drug‐related side effects and contributing risk factors was defined as a mean value of 3.75 or higher, corresponding to at least 75% agreement among participants [16].

To examine any statistically significant differences between sex, age, work experience and the specialists' opinions about drug‐related side effects and risk factors, Student's *t*‐tests were conducted. Sex comparisons were made between male and female respondents; age was dichotomized into ≤ 40 years and > 40 years; and work experience into ≤ 10 years and > 10 years categories. The statistically significant difference was set at *p* < 0.05. All analyses were conducted using SPSS version 22 (IBM Corp., Armonk, NY, USA). The study followed the Guidelines for Reporting of Statistics for Clinical Research in Urology [17] and adhered to the Statistical Analyses and Methods in the Published Literature (SAMPL) guidelines [18] to ensure transparent reporting.

### 2.7 Ethical Considerations

Ethics approval for this study was obtained from the Ethics Committee of Iran University of Medical Sciences (IR.IUMS.REC.1401.1007). All participants were informed of the study objectives and procedures before participation, and written informed consents were obtained from all participants before completing the questionnaires. Participation was entirely voluntary, and respondents had the right to withdraw at any time. To ensure confidentiality, all responses were anonymized, and no identifiable personal information was collected. Data were securely stored and accessible only to the research team.

## 3 Results

### 3.1 Participant Characteristics

As presented in Table 1, a total of 20 specialists (62.5%) participated in this study, comprising 16 pediatric cardiologists and 4 pediatric cardiology fellows. The sample consisted of 65% male and 35% female participants, with an average age of 45.5 years (SD = 10.3; range: 33–64 years) and a mean clinical experience of 16.5 years (SD = 8.4; range: 6–32 years).

**Table 1:** Demographic characteristics of the participants.

Characteristics	Fr (%)
Sex	
Male	13 (65)
Female	7 (35)
Age (years)	(Mean ± SD = 45.5 ± 10.3, range = 33–64)
≤ 40 years	9 (45)
> 40 years	11 (55)
Specialty	
Pediatric cardiologist	16 (80)
Pediatric cardiology fellow	4 (20)
Work experience (years)	(Mean ± SD = 16.5 ± 8.4)
≤ 10 years	8 (40)
> 10 years	12 (60)

### 3.2 Drug‐Related Side Effects

This study investigated commonly prescribed medications for pediatric patients with CHD and identified reported side effects. Table 2 provides an overview of these medications and their associated side effects according to specialists' opinions.

**Table 2:** Drug‐related side effects in children with CHD.

No.	Drug name/prescription status (Fr, %)	Side effect	Strongly disagree Fr (%)	Disagree Fr (%)	Neither agree nor disagree Fr (%)	Agree Fr (%)	Strongly agree Fr (%)	Mean ± SD
1	Acetaminophen (20, 100)	Hyponatremia	0	0	7 (35)	9 (45)	4 (20)	2.15 ± 0.75
		Patent ductus arteriosus closure	3 (15)	4 (20)	10 (50)	3 (15)	0	3.35 ± 0.93
		Thrombocytopenia	1 (5)	0	6 (30)	9 (45)	4 (20)	2.25 ± 0.97
2	Amiodarone (20, 100)	Hypotension	30 (6)	50 (10)	15 (3)	5 (1)	0	4.05 ± 0.83*
		Bradycardia	30 (6)	40 (8)	30 (6)	0 (0)	0	4 ± 0.79*
		Ventricular dysfunction	25 (5)	25 (5)	25 (5)	25 (5)	0	3.5 ± 1.15
		Intermittent sinus	15 (3)	20 (4)	50 (10)	15 (3)	0	3.35 ± 0.93
		Hypothyroidism	40 (8)	60 (12)	0 (0)	0 (0)	0	4.4 ± 0.5*
		Anemia	15 (3)	10 (2)	60 (12)	15 (3)	0	3.25 ± 0.91
		Occasional junctional escape beats	15 (3)	20 (4)	50 (10)	15 (3)	0	3.35 ± 0.93
		Leukopenia	20 (4)	15 (3)	50 (10)	15 (3)	0	3.4 ± 0.99
		Hyperemic mucosa	0 (0)	15 (3)	70 (14)	5 (1)	10 (2)	2.9 ± 0.79
		Haziness	15 (3)	20 (4)	40 (8)	25 (5)	0	3.25 ± 1.02
		Ground glass opacities	10 (2)	25 (5)	50 (10)	15 (3)	0	3.3 ± 0.86
		Pulmonary edema	15 (3)	20 (4)	40 (8)	25 (5)	0	3.25 ± 1.02
		Inflamed with brown secretions	5 (1)	15 (3)	70 (14)	10 (2)	0	3.15 ± 0.67
		Hypoxia	5 (1)	10 (2)	50 (10)	35 (7)	0	2.85 ± 0.81
3	Aspirin (20, 100)	Hematochezia	30 (6)	35 (7)	25 (5)	10 (2)	0	3.85 ± 0.99*
		Thrombotic stroke, right atrial thrombus	5 (1)	5 (1)	30 (6)	60 (12)	0	2.55 ± 0.83
		Cardiac tamponade	5 (1)	20 (4)	30 (6)	45 (9)	0	2.85 ± 0.93
		Pulmonary hemorrhage	0 (0)	25 (5)	50 (10)	25 (5)	0	3 ± 0.73
		Bleeding from pseudoaneurysm	10 (2)	30 (6)	35 (7)	25 (5)	0	3.25 ± 0.97
		Bleeding from the central line	0 (0)	5 (1)	25 (5)	55 (11)	15 (3)	2.2 ± 0.77
4	Atenolol (8, 40)	Rash	0 (0)	50 (4)	37 (3)	12 (1)	0 (0)	3.38 ± 0.74
		Sinus bradycardia	0 (0)	25 (2)	12 (1)	62 (5)	0 (0)	2.63 ± 0.92
		Palpitations	37 (3)	62 (5)	0 (0)	0 (0)	0 (0)	4.38 ± 0.52*
		Asthma	37 (3)	37 (3)	25 (2)	0 (0)	0 (0)	4.13 ± 0.83*
		Vertigo	25 (2)	62 (5)	0 (0)	12 (1)	0 (0)	4 ± 0.93*
		Headache	25 (2)	0 (0)	37 (3)	37 (3)	0 (0)	3.13 ± 1.25
		Asthenia	25 (2)	12 (1)	37 (3)	25 (2)	0 (0)	3.38 ± 1.19
		Leg pain	12 (1)	25 (2)	37 (3)	25 (2)	0 (0)	3.25 ± 1.04
		Cold extremities	37 (3)	37 (3)	12 (1)	12 (1)	0 (0)	4 ± 1.07*
		Epistaxis	0 (0)	0 (0)	12 (1)	75 (6)	12 (1)	2 ± 0.53
		Gynecomastia	0 (0)	12 (1)	37 (3)	50 (4)	0 (0)	2.63 ± 0.74
5	Bosentan (20, 100)	Hemolytic anemia	5 (1)	20 (4)	65 (13)	10 (2)	0 (0)	3.2 ± 0.7
		Fever	20 (4)	10 (2)	55 (11)	15 (3)	0 (0)	3.35 ± 0.99
6	Captopril (20, 100)	Hypotension	30 (6)	40 (8)	20 (4)	10 (2)	0 (0)	3.9 ± 0.97*
		Poor feeding	5 (1)	15 (3)	30 (6)	50 (10)	0 (0)	2.75 ± 0.91
		Tachycardia	0 (0)	30 (6)	25 (5)	45 (9)	0 (0)	2.85 ± 0.88
		Acute renal failure	15 (3)	45 (9)	30 (6)	10 (2)	0 (0)	3.65 ± 0.88
		Oliguria	5 (1)	50 (10)	20 (4)	25 (5)	0 (0)	3.35 ± 0.93
		Oxygen saturation deficit	5 (1)	10 (2)	50 (10)	35 (7)	0 (0)	2.85 ± 0.81
		Weight loss	0 (0)	15 (3)	35 (7)	50 (10)	0 (0)	2.65 ± 0.75
		Rhinorrhea	5 (1)	0 (0)	60 (12)	35 (7)	0 (0)	2.75 ± 0.72
7	Dexmedetomidine(8, 40)	Hypotension	0 (0)	62 (5)	25 (2)	12 (1)	0 (0)	3.5 ± 0.76
		Bradycardia	12 (1)	50 (4)	37 (3)	0 (0)	0 (0)	3.75 ± 0.71*
		Tachycardia	0 (0)	0 (0)	62 (5)	37 (3)	0 (0)	2.63 ± 0.52
		Arrhythmias	0 (0)	12 (1)	75 (6)	12 (1)	0 (0)	3 ± 0.53
		Cardiac ischemia	0 (0)	0 (0)	75 (6)	25 (2)	0 (0)	2.75 ± 0.46
		Cardiac and cardiopulmonary arrest	0 (0)	25 (2)	62 (5)	12 (1)	0 (0)	3.13 ± 0.64
		Deep stimulation	0 (0)	25 (2)	50 (4)	25 (2)	0 (0)	3 ± 0.76
		Nausea	0 (0)	50 (4)	50 (4)	0 (0)	0 (0)	3.5 ± 0.53
		Vomiting	12 (1)	50 (4)	25 (2)	12 (1)	0 (0)	3.63 ± 0.92
		hypopnea	0 (0)	25 (2)	50 (4)	25 (2)	0 (0)	3 ± 0.76
8	Digoxin (20, 100)	Hyperkalemia	15 (3)	25 (5)	25 (5)	30 (6)	5 (1)	3.6 ± 1.19
		Severe cardiac arrhythmias	35 (7)	25 (5)	10 (2)	30 (6)	0 (0)	3.65 ± 1.27
		Hemodynamic instability	15 (3)	25 (5)	25 (5)	30 (6)	5 (1)	3.15 ± 1.18
9	Enalapril (18, 90)	Rash	22 (4)	27 (5)	38 (7)	11 (2)	0 (0)	3.61 ± 0.98
		Vertigo	11 (2)	27 (5)	55 (10)	5 (1)	0 (0)	3.44 ± 0.78
		Headache	16 (3)	44 (8)	27 (5)	11 (2)	0 (0)	3.67 ± 0.91
		Asthenia	11 (2)	5 (1)	72 (13)	11 (2)	0 (0)	3.17 ± 0.79
10	Flecainide (17, 85)	Ventricular dysfunction	35 (6)	23 (4)	29 (5)	11 (2)	0 (0)	3.82 ± 1.07*
		Inadvertent overdose	5 (1)	17 (3)	58 (10)	17 (3)	0 (0)	3.12 ± 0.78
		Bundle branch block	17 (3)	17 (3)	47 (8)	17 (3)	0 (0)	3.35 ± 1
11	Furosemide (20, 100)	Hypokalemia	60 (12)	30 (6)	10 (2)	0 (0)	0 (0)	4.5 ± 0.69*
		Hypocalcemia	10 (2)	10 (2)	40 (8)	40 (8)	0 (0)	2.9 ± 0.97
		Hypovolemia	35 (7)	55 (11)	5 (1)	5 (1)	0 (0)	4.2 ± 0.77*
		Bone fractures	35 (7)	45 (9)	20 (4)	0 (0)	0 (0)	4.15 ± 0.75*
12	Heparin (20, 100)	Thrombocytopenia	50 (10)	40 (8)	10 (2)	0 (0)	0 (0)	4.4 ± 0.68*
		Postoperative bleeding	55 (11)	30 (6)	15 (3)	0 (0)	0 (0)	4.4 ± 0.75*
13	Ibuprofen (17, 85)	Necrotizing enterocolitis	23 (4)	58 (10)	11 (2)	5 (1)	0 (0)	4 ± 0.79*
		Bronchopulmonary dysplasia	11 (2)	5 (1)	58 (10)	23 (4)	0 (0)	3.06 ± 0.9
		Tachypnoea	23 (4)	52 (9)	5 (1)	17 (3)	0 (0)	3.82 ± 1.01*
		Oliguria	17 (3)	52 (9)	23 (4)	5 (1)	0 (0)	3.82 ± 0.81*
		Acute kidney injury	0 (0)	0 (0)	47 (8)	52 (9)	0 (0)	2.47 ± 0.51
		Retinopathy of prematurity	17 (3)	35 (6)	29 (5)	17 (3)	0 (0)	3.76 ± 0.97*
		Intraventricular hemorrhage	23 (4)	41 (7)	23 (4)	11 (2)	0 (0)	4.35 ± 0.7*
		Gastrointestinal bleed	47 (8)	41 (7)	11 (2)	0 (0)	0 (0)	4.35 ± 0.7*
		Pulmonary hemorrhage	11 (2)	29 (5)	41 (7)	17 (3)	0 (0)	3.35 ± 0.93
14	Indomethacin (10, 50)	Necrotizing enterocolitis	31 (5)	25 (4)	0 (0)	6 (1)	0 (0)	4.3 ± 0.95*
		Gastrointestinal perforation	31 (5)	25 (4)	6 (1)	0 (0)	0 (0)	4.4 ± 0.7*
		Bronchopulmonary dysplasia	6 (1)	0 (0)	43 (7)	12 (2)	0 (0)	3 ± 0.82
		Oliguria	25 (4)	25 (4)	12 (2)	0 (0)	0 (0)	4.2 ± 0.79*
		Anuria	31 (5)	18 (3)	12 (2)	0 (0)	0 (0)	4.3 ± 0.82*
		Gastrointestinal hemorrhage	18 (3)	25 (4)	18 (3)	0 (0)	0 (0)	4 ± 0.82*
		Intracerebral hemorrhage	18 (3)	18 (3)	25 (4)	0 (0)	0 (0)	3.9 ± 0.88*
		Hyponatremia	12 (2)	12 (2)	31 (5)	6 (1)	0 (0)	3.5 ± 0.97
		Elevation of serum creatinine	25 (4)	37 (6)	0 (0)	0 (0)	0 (0)	4.4 ± 0.52*
		Patent ductus arteriosus closure	6 (1)	0 (0)	43 (7)	12 (2)	0 (0)	3 ± 0.82
		Thrombocytopenia	31 (5)	18 (3)	6 (1)	6 (1)	0 (0)	4.2 ± 1.03*
15	Iloprost (11, 55)	Wheezing	9 (1)	18 (2)	45 (5)	27 (3)	0 (0)	3.2 ± 0.92
		Facial flushing	9 (1)	18 (2)	45 (5)	27 (3)	0 (0)	3.7 ± 0.82*
16	Sildenafil (20, 100)	Restlessness	10 (2)	30 (6)	50 (10)	10 (2)	0 (0)	3.4 ± 0.82
		Rhinorrhea	5 (1)	20 (4)	65 (13)	10 (2)	0 (0)	3.2 ± 0.7
		Facial flushes	15 (3)	50 (10)	30 (6)	5 (1)	0 (0)	3.75 ± 0.79*
17	Sotalol (17, 85)	Bradycardia	41 (7)	58 (10)	0 (0)	0 (0)	0 (0)	4.41 ± 0.51*
		Eye deviation	17 (3)	11 (2)	52 (9)	17 (3)	0 (0)	3.29 ± 0.99
		Nystagmus	11 (2)	5 (1)	76 (13)	5 (1)	0 (0)	3.24 ± 0.75
		Lethargy	11 (2)	35 (6)	52 (9)	0 (0)	0 (0)	3.59 ± 0.71
		Irritability	11 (2)	35 (6)	47 (8)	5 (1)	0 (0)	3.53 ± 0.8
		Hypothermia	11 (2)	35 (6)	52 (9)	0 (0)	0 (0)	3.59 ± 0.71
		Left‐sided hypertonicity	0 (0)	11 (2)	70 (12)	17 (3)	0 (0)	2.94 ± 0.56
18	Tadalafil (16, 80)	Headache	12 (2)	75 (12)	12 (2)	0 (0)	0 (0)	4 ± 0.52*
		Myalgia	0 (0)	50 (8)	43 (7)	6 (1)	0 (0)	3.44 ± 0.63
19	Prostaglandin E1 (18, 90)	Gastric outlet obstruction syndrome	5 (1)	11 (2)	61 (11)	22 (4)	0 (0)	3 ± 0.77
		Vascular fragility	5 (1)	16 (3)	72 (13)	5 (1)	0 (0)	3.22 ± 0.65
		Electrolyte imbalance	5 (1)	33 (6)	55 (10)	5 (1)	0 (0)	3.39 ± 0.7
		Metabolic alkalosis	11 (2)	33 (6)	38 (7)	16 (3)	0 (0)	3.39 ± 0.92
		Bradycardia	5 (1)	38 (7)	44 (8)	11 (2)	0 (0)	3.39 ± 0.78
		Pulmonary hypertension	5 (1)	27 (5)	33 (6)	33 (6)	0 (0)	3 ± 0.97
		Cardiac arrhythmia	5 (1)	33 (6)	27 (5)	33 (6)	0 (0)	3.06 ± 0.94
		Tachycardia	16 (3)	33 (6)	16 (3)	33 (6)	0 (0)	3.11 ± 0.96
		Cardiac arrest	0 (0)	22 (4)	50 (9)	27 (5)	0 (0)	3.33 ± 1.14
		Elongated and thickened pyloric musculature	0 (0)	16 (3)	55 (10)	27 (5)	0 (0)	2.94 ± 0.73
		Periosteal thickening in the femurs and humerus, radius and ulna, tibias, distal portions of the tibia, and fibulae	0 (0)	11 (2)	66 (12)	22 (4)	0 (0)	2.89 ± 0.68
		Cortical hyperostosis	16 (3)	44 (8)	22 (4)	16 (3)	0 (0)	2.89 ± 0.58
		Respiratory insufficiency	0 (0)	0 (0)	61 (11)	38 (7)	0 (0)	3.61 ± 0.98
		Chylothorax	0 (0)	5 (1)	50 (9)	44 (8)	0 (0)	2.61 ± 0.5
		Apnea	55 (10)	38 (7)	5 (1)	0 (0)	0 (0)	4.5 ± 0.62*
		Hypoventilation	33 (6)	50 (9)	16 (3)	0 (0)	0 (0)	4.17 ± 0.71*
		Nasal cannula	11 (2)	11 (2)	50 (9)	16 (3)	11 (2)	2.94 ± 1.11
		Pain on manipulation	5 (1)	11 (2)	66 (12)	11 (2)	5 (1)	3 ± 0.84
		Pain during handling	5 (1)	11 (2)	61 (11)	16 (3)	5 (1)	2.94 ± 0.87
		Edema	11 (2)	27 (5)	44 (8)	16 (3)	0 (0)	3.33 ± 0.91
		Muscle stiffness	0 (0)	11 (2)	50 (9)	38 (7)	0 (0)	2.72 ± 0.67
		Fever	33 (6)	38 (7)	16 (3)	11 (2)	0 (0)	3.94 ± 1*
		Irritability	11 (2)	33 (6)	38 (7)	16 (3)	0 (0)	3.39 ± 0.92
		Desaturation	27 (5)	5 (1)	38 (7)	27 (5)	0 (0)	3.33 ± 1.19
		Hyperthermia	38 (7)	27 (5)	33 (6)	0 (0)	0 (0)	4.06 ± 0.87*
		Flushing	33 (6)	44 (8)	22 (4)	0 (0)	0 (0)	4.11 ± 0.76*
		Convulsions	16 (3)	27 (5)	50 (9)	5 (1)	0 (0)	3.56 ± 0.86
		Diarrhea	16 (3)	27 (5)	38 (7)	16 (3)	0 (0)	3.44 ± 0.98
		Non‐bilious vomiting	5 (1)	22 (4)	55 (10)	16 (3)	0 (0)	3.17 ± 0.79
		Polyuria	0 (0)	16 (3)	55 (10)	27 (5)	0 (0)	2.89 ± 0.68
		Disseminated intravascular coagulation	11 (2)	11 (2)	61 (11)	16 (3)	0 (0)	3.17 ± 0.86

* Indicated as highly approved drug‐related side effects.

There was strong consensus among participants regarding certain drug‐related side effects. For instance, hypotension was noted as a severe side effect of Amiodarone (4.05 ± 0.83), with 80% of respondents agreeing or strongly agreeing. Similarly, bradycardia was frequently associated with Dexmedetomidine (3.75 ± 0.71), and hypotension was associated with Atenolol (4.38 ± 0.52).

Additional reported side effects revealed significant safety concerns across various prescribed CHD medications in pediatric patients, with hepatic and metabolic complications. Acetaminophen was commonly associated with liver toxicity and drowsiness, while Amiodarone presented risks of bone marrow suppression, hepatic and pulmonary fibrosis, hypothyroidism, and severe hypotension during infusion. Aspirin therapy, particularly at high doses, demonstrated associations with gastrointestinal issues, Reye's syndrome, and bleeding disorders.

Atenolol and Bosentan were linked to metabolic disturbances, hypotension, and elevated liver enzymes, indicating the importance of close hepatic and metabolic monitoring. Captopril and Enalapril were noted for causing respiratory and electrolyte imbalance side effects, with captopril additionally being associated with hyperkalemia and persistent cough. Digoxin was connected with toxic arrhythmias and electrolyte imbalance, while flecainide had risks of causing arrhythmic issues, particularly in neonates. Dexmedetomidine, Furosemide, and Sildenafil were associated with fluid retention, hypotension, and electrolyte disturbances.

These insights highlight the importance of personalized side effect monitoring, particularly for hepatic, cardiac, and metabolic parameters in pediatric CHD patients. Table 3 details all medication‐associated side effects reported by study participants.

**Table 3:** Additional reported drug‐related side effects in children with CHD.

Drug name	Reported side effects (number of participants)
Acetaminophen	Liver toxicity (4), liver failure (3), drowsiness (1), enzyme elevation at high doses (2)
Amiodarone	Bone marrow suppression (1), skin rash resembling lupus (1), hepatic fibrosis (2), hypothyroidism (2), severe hypotension during infusion (1)
Aspirin	Gastrointestinal issues (2), Reye's syndrome (3), Petechiae and gum bleeding (1), platelet aggregation disorders (1)
Atenolol	Poor diabetes control (1), confusion (2), blurred vision (2), sweating (2), hypotension (2)
Bosentan	Gastrointestinal symptoms (2), elevated liver enzymes (3), hypotension (2)
Captopril	Chronic cough (4), hyperkalemia (2), increased respiratory secretions (1), hypotension (2)
Dexmedetomidine	Allergic reactions (1), urinary volume changes (1), weight loss (1)
Digoxin	Gastrointestinal disturbances (2), vision disturbances (1), bradycardia (2), toxic dose arrhythmias (3)
Enalapril	Dry cough (2), hypotension (3), blurred vision (1), headache (1)
Flecainide	QT prolongation (2), potential arrhythmias (2), hypotension (1)
Furosemide	Electrolyte disturbances (2), alkalosis (1), hearing loss in neonates (1)
Heparin	Chest pain (1), shortness of breath (1), fever (1)
Ibuprofen	Stomach discomfort (1), constipation (1), drowsiness (1)
Indomethacin	Stomach irritation (1), drowsiness (1), headache (1)
Iloprost	Hypotension (1), itching (1), dizziness (1)
Sildenafil	Hypotension (3), blurred vision (3), chest pain (3)
Sotalol	Bradycardia (2), QT prolongation (2), gastrointestinal issues (1)
Tadalafil	Hypotension (1), priapism (1)
Prostaglandin E1	Apnea (1)

### 3.3 Contributing Risk Factors for Drug‐Related Side Effects

Table 4 shows the risk factors for drug‐related side effects in pediatric patients with CHD. Among these risk factors, dosage emerged as particularly significant, especially for medications such as Digoxin (4.7 + 0.47) and Heparin (4.65 + 0.49).

**Table 4:** Risk factors associated with drug‐related side effects in children with CHD.

No.	Drug name/prescription status (Fr, %)	Risk factors associated with drug‐related side effects	Disagree Fr (%)	Neither agree nor disagree Fr (%)	Agree Fr (%)	Strongly agree Fr (%)	Mean ± SD
1	Acetaminophen (20, 100)	Drug dose	0 (0)	1 (5)	12 (60)	7 (35)	4.3 ± 0.57*
		Age	1 (5)	4 (20)	11 (55)	4 (20)	3.8 ± 0.89*
		Weight	1 (5)	7 (35)	8 (40)	4 (20)	3.65 ± 0.93
		Sex	5 (1)	50 (10)	40 (8)	5 (1)	2.45 ± 0.6
		Polypharmacy	0 (0)	7 (35)	12 (60)	1 (5)	3.7 ± 0.57
		Comorbidities	1 (5)	5 (25)	12 (60)	2 (10)	3.75 ± 0.72*
2	Amiodarone (20, 100)	Drug dose	0 (0)	0 (0)	9 (45)	11 (55)	4.55 ± 0.51*
		Age	1 (5)	0 (0)	14 (70)	5 (25)	4.15 ± 0.67*
		Weight	3 (15)	4 (20)	9 (45)	4 (20)	3.7 ± 0.98
		Sex	8 (40)	12 (60)	0 (0)	0 (0)	2.6 ± 0.5
		Polypharmacy	0 (0)	2 (10)	10 (50)	8 (40)	4.3 ± 0.66*
		Comorbidities	0 (0)	0 (0)	10 (50)	10 (50)	4.5 ± 0.51*
3	Aspirin (20, 100)	Drug dose	0 (0)	0 (0)	9 (45)	11 (55)	4.55 ± 0.51*
		Age	0 (0)	6 (30)	7 (35)	7 (35)	4.05 ± 0.83*
		Weight	1 (5)	8 (40)	5 (25)	6 (30)	3.8 ± 0.95*
		Sex	8 (40)	12 (60)	0 (0)	0 (0)	2.6 ± 0.5
		Polypharmacy	0 (0)	2 (10)	12 (60)	6 (30)	4.2 ± 0.62*
		Comorbidities	2 (10)	2 (10)	11 (55)	5 (25)	3.95 ± 0.89*
4	Atenolol (8, 40)	Drug dose	0 (0)	0 (0)	3 (37)	5 (62)	4.63 ± 0.52*
		Age	1 (12)	2 (25)	3 (37)	2 (25)	3.75 ± 1.04*
		Weight	2 (25)	2 (25)	2 (25)	2 (25)	3.5 ± 1.2
		Sex	5 (62)	3 (37)	0 (0)	0 (0)	2.38 ± 0.52
		Polypharmacy	0 (0)	0 (0)	4 (50)	4 (50)	4.5 ± 0.53*
		Comorbidities	3 (37)	1 (12)	2 (25)	2 (25)	3.38 ± 1.3
5	Bosentan (20, 100)	Drug dose	0 (0)	3 (15)	11 (55)	6 (30)	4.15 ± 0.67*
		Age	0 (0)	3 (15)	10 (50)	7 (35)	4.2 ± 0.7*
		Weight	0 (0)	5 (25)	10 (50)	5 (25)	4 ± 0.73*
		Sex	8 (40)	12 (60)	0 (0)	0 (0)	2.6 ± 0.5
		Polypharmacy	0 (0)	5 (25)	10 (50)	5 (25)	4 ± 0.73*
		Comorbidities	1 (5)	2 (10)	12 (60)	5 (25)	4.05 ± 0.76*
6	Captopril (20, 100)	Drug dose	0 (0)	0 (0)	10 (50)	10 (50)	4.5 ± 0.51*
		Age	1 (5)	2 (10)	10 (50)	7 (35)	4.15 ± 0.81*
		Weight	2 (10)	6 (30)	7 (35)	5 (25)	3.75 ± 0.97*
		Sex	8 (40)	10 (50)	1 (5)	1 (5)	2.75 ± 0.79
		Polypharmacy	0 (0)	2 (10)	12 (60)	6 (30)	4.2 ± 0.62*
		Comorbidities	0 (0)	2 (10)	10 (50)	8 (40)	4.3 ± 0.66*
7	Dexmedetomidine (8, 40)	Drug dose	0 (0)	1 (12)	3 (37)	4 (50)	4.38 ± 0.74*
		Age	0 (0)	1 (12)	4 (50)	3 (37)	4.25 ± 0.71*
		Weight	0 (0)	2 (25)	3 (37)	3 (37)	4.13 ± 0.83*
		Sex	3 (37)	5 (62)	0 (0)	0 (0)	2.63 ± 0.52
		Polypharmacy	0 (0)	1 (12)	5 (62)	2 (25)	4.13 ± 0.64*
		Comorbidities	0 (0)	3 (37)	3 (37)	2 (25)	3.88 ± 0.83*
8	Digoxin (20, 100)	Drug dose	0 (0)	0 (0)	6 (30)	14 (70)	4.7 ± 0.47*
		Age	1 (5)	1 (5)	8 (40)	10 (50)	4.35 ± 0.81*
		Weight	3 (15)	2 (10)	11 (55)	4 (20)	3.8 ± 0.95*
		Sex	10 (50)	10 (50)	0 (0)	0 (0)	2.5 ± 0.51
		Polypharmacy	1 (5)	0 (0)	11 (55)	8 (40)	4.3 ± 0.73*
		Comorbidities	1 (5)	1 (5)	11 (55)	7 (35)	4.2 ± 0.77*
9	Enalapril (18, 90)	Drug dose	0 (0)	1 (5)	7 (38)	10 (55)	4.5 ± 0.62*
		Age	1 (5)	3 (16)	8 (44)	6 (33)	4.06 ± 0.87*
		Weight	2 (11)	5 (27)	8 (44)	3 (16)	3.67 ± 0.91
		Sex	8 (44)	10 (55)	0 (0)	0 (0)	2.56 ± 0.51
		Polypharmacy	0 (0)	3 (16)	9 (50)	6 (33)	4.17 ± 0.71*
		Comorbidities	0 (0)	4 (22)	5 (27)	9 (50)	4.28 ± 0.83*
10	Flecainide (17, 85)	Drug dose	0 (0)	0 (0)	8 (47)	9 (52)	4.53 ± 0.51*
		Age	2 (11)	2 (11)	6 (35)	7 (41)	4.06 ± 1.03*
		Weight	4 (23)	2 (11)	7 (41)	4 (23)	3.65 ± 1.11
		Sex	7 (41)	10 (58)	0 (0)	0 (0)	2.59 ± 0.51
		Polypharmacy	0 (0)	0 (0)	10 (58)	7 (41)	4.41 ± 0.51*
		Comorbidities	1 (5)	2 (11)	9 (52)	5 (29)	4.06 ± 0.83*
11	Furosemide (20, 100)	Drug dose	0 (0)	0 (0)	8 (40)	12 (60)	4.6 ± 0.5*
		Age	2 (10)	1 (5)	9 (45)	8 (40)	4.15 ± 0.93*
		Weight	3 (15)	6 (30)	8 (40)	3 (15)	3.55 ± 0.94
		Sex	11 (55)	8 (40)	1 (5)	0 (0)	2.5 ± 0.61
		Polypharmacy	0 (0)	0 (0)	11 (55)	9 (45)	4.45 ± 0.51*
		Comorbidities	1 (5)	0 (0)	11 (55)	8 (40)	4.3 ± 0.73*
12	Heparin (20, 100)	Drug dose	0 (0)	0 (0)	7 (35)	13 (65)	4.65 ± 0.49*
		Age	1 (5)	5 (25)	9 (45)	5 (25)	3.9 ± 0.85*
		Weight	1 (5)	5 (25)	10 (50)	4 (20)	3.85 ± 0.81*
		Sex	9 (45)	11 (55)	0 (0)	0 (0)	2.55 ± 0.51
		Polypharmacy	0 (0)	3 (15)	9 (45)	8 (40)	4.25 ± 0.72*
		Comorbidities	1 (5)	3 (15)	7 (35)	9 (45)	4.2 ± 0.89*
13	Ibuprofen (17, 85)	Drug dose	0 (0)	0 (0)	7 (41)	10 (58)	4.59 ± 0.51*
		Age	0 (0)	0 (0)	7 (41)	10 (58)	4.59 ± 0.51*
		Weight	0 (0)	1 (5)	9 (52)	7 (41)	4.35 ± 0.61*
		Sex	8 (47)	8 (47)	0 (0)	1 (5)	2.65 ± 0.79
		Polypharmacy	0 (0)	1 (5)	8 (47)	8 (47)	4.41 ± 0.62*
		Comorbidities	0 (0)	0 (0)	7 (41)	10 (58)	4.59 ± 0.51*
14	Indomethacin (10, 50)	Drug dose	0 (0)	0 (0)	4 (40)	6 (60)	4.6 ± 0.52*
		Age	0 (0)	0 (0)	6 (60)	4 (40)	4.4 ± 0.52*
		Weight	0 (0)	2 (20)	6 (60)	2 (20)	4 ± 0.67*
		Sex	3 (30)	5 (50)	1 (10)	1 (10)	3 ± 0.94
		Polypharmacy	0 (0)	1 (10)	4 (40)	5 (50)	4.4 ± 0.7*
		Comorbidities	0 (0)	1 (10)	4 (40)	5 (50)	4.4 ± 0.7*
15	Iloprost (11, 55)	Drug dose	0 (0)	0 (0)	5 (45)	6 (54)	4.55 ± 0.52*
		Age	0 (0)	0 (0)	7 (63)	4 (36)	4.36 ± 0.5*
		Weight	0 (0)	2 (18)	6 (54)	3 (27)	4.09 ± 0.7*
		Sex	5 (45)	6 (54)	0 (0)	0 (0)	2.55 ± 0.52
		Polypharmacy	0 (0)	2 (18)	6 (54)	3 (27)	4.09 ± 0.7*
		Comorbidities	0 (0)	1 (9)	7 (63)	3 (27)	4.18 ± 0.6*
16	Sildenafil (20, 100)	Drug dose	0 (0)	1 (5)	11 (55)	8 (40)	4.35 ± 0.59*
		Age	1 (5)	5 (25)	9 (45)	5 (25)	3.9 ± 0.85*
		Weight	2 (10)	7 (35)	9 (45)	2 (10)	3.55 ± 0.83
		Sex	8 (40)	8 (40)	2 (10)	2 (10)	2.9 ± 0.97
		Polypharmacy	1 (5)	3 (15)	8 (40)	8 (40)	4.15 ± 0.88*
		Comorbidities	1 (5)	5 (25)	7 (35)	7 (35)	4 ± 0.92*
17	Sotalol (17, 85)	Drug dose	0 (0)	0 (0)	10 (58)	7 (41)	4.41 ± 0.51*
		Age	2 (11)	1 (5)	9 (52)	5 (29)	4 ± 0.94*
		Weight	3 (17)	4 (23)	7 (41)	3 (17)	3.59 ± 1
		Sex	7 (41)	10 (58)	0 (0)	0 (0)	2.59 ± 0.51
		Polypharmacy	0 (0)	3 (17)	8 (47)	6 (35)	4.18 ± 0.73*
		Comorbidities	1 (5)	3 (17)	7 (41)	6 (35)	4.06 ± 0.9*
18	Tadalafil (16, 80)	Drug dose	0 (0)	2 (12)	8 (50)	6 (37)	4.25 ± 0.68*
		Age	1 (6)	2 (12)	8 (50)	5 (31)	4.06 ± 0.85*
		Weight	2 (12)	6 (37)	5 (31)	3 (18)	3.56 ± 0.96
		Sex	6 (37)	7 (43)	2 (12)	1 (6)	2.88 ± 0.89
		Polypharmacy	0 (0)	3 (18)	7 (43)	6 (37)	4.19 ± 0.75*
		Comorbidities	1 (6)	4 (25)	7 (43)	4 (25)	3.88 ± 0.89*
19	Prostaglandin E1 (18, 90)	Drug dose	0 (0)	0 (0)	7 (38)	11 (61)	4.61 ± 0.5*
		Age	1 (5)	4 (22)	6 (33)	7 (38)	4.06 ± 0.94*
		Weight	0 (0)	3 (16)	8 (44)	7 (38)	4.22 ± 0.73*
		Sex	8 (44)	10 (55)	0 (0)	0 (0)	2.56 ± 0.51
		Polypharmacy	1 (5)	5 (27)	6 (33)	6 (33)	3.94 ± 0.94*
		Comorbidities	1 (5)	3 (16)	8 (44)	6 (33)	4.06 ± 0.87*

* Indicated as highly approved risk factors associated with drug‐related side effects.

Polypharmacy was also identified as a significant risk factor, especially for Amiodarone (4.3 + 0.66) and Furosemide (4.45 + 0.51), highlighting the complexity of medication management in pediatric patients with CHD requiring multiple drugs. Since none of the participants selected “Strongly Disagree” for any of the listed risk factors, this response category was excluded from the table.

### 3.4 Significant Difference Between Demographic Variables and Drug‐Related Outcomes

The *t*‐test analysis revealed significant differences between the participants' demographic characteristics (sex, age, and work experience) and their perspectives on drug‐related side effects and risk factors. The results showed that there was a statistically significant difference between participants' sex and the overall drug‐related side effects (*p* = 0.035) and drug risk factors (*p* = 0.013), suggesting the influence of potential sex variations in professional assessment. Atenolol‐related cold extremities (*p* = 0.035), Indomethacin‐related necrotizing enterocolitis (*p* = 0.001), and Prostaglandin E1‐related electrolyte imbalance (*p* = 0.037) were perceived more by the specialists aged > 40 years old. Similarly, statistically significant differences emerged between specialists with > 10 years versus those with ≤ 10 years of work experience regarding Enalapril‐related risk factors (*p* = 0.002), Heparin‐related risk factors (*p* = 0.003), and Prostaglandin E1‐related electrolyte imbalance (*p* = 0.002). Table 5 shows these statistically significant differences between demographic variables and drug‐related outcomes.

**Table 5:** Statistically significant difference between demographic variables and drug‐related outcomes.

Drug	Outcome		Sex (male vs. female)	Age (≤ 40 vs. > 40)	Work experience (≤ 10 vs. > 10)
Overall	Drug side effects		*t* = 2.34, *p* = 0.035*	*t* = −3.66, *p* = 0.035*	*t* = −4.20, *p* < 0.001**
Overall	Drug risk factors		*t* = 2.87, *p* = 0.013*	*t* = −4.50, *p* < 0.001**	*t* = −5.12, *p* < 0.001**
Amiodarone	Drug‐related side effects	Hypothyroidism	*t* = 2.21, *p* = 0.022*	*t* = 1.45, *p* = 0.152	*t* = 1.20, *p* = 0.234
Aspirin		Pulmonary hemorrhage	*t* = 2.12, *p* = 0.033*	*t* = −1.98, *p* = 0.058	*t* = −2.05, *p* = 0.051
Atenolol		Cold extremities	*t* = 1.56, *p* = 0.189	*t* = −3.66, *p* = 0.035*	*t* = −3.66, *p* = 0.035*
Ibuprofen		Intraventricular hemorrhage	*t* = 2.34, *p* = 0.018*	*t* = −2.66, *p* = 0.048*	*t* = −2.08, *p* = 0.059
Indomethacin		Necrotizing enterocolitis	*t* = 1.45, *p* = 0.105	*t* = −5.00, *p* = 0.001**	*t* = −5.00, *p* = 0.001**
Sotalol		Bradycardia	*t* = 1.32, *p* = 0.250	*t* = −2.38, *p* = 0.034*	*t* = −1.95, *p* = 0.086
Prostaglandin E1		Electrolyte imbalance	*t* = 0.87, *p* = 0.446	*t* = −2.29, *p* = 0.037*	*t* = −1.30, *p* = 0.212
Aspirin	Risk factors	Comorbidities	*t* = 0.78, *p* = 0.454	*t* = −3.12, *p* = 0.009**	*t* = −2.89, *p* = 0.004**
Iloprost		Age	*t* = 0.65, *p* = 0.391	*t* = −2.83, *p* = 0.030*	*t* = −2.65, *p* = 0.033*
Sildenafil		Weight	*t* = 0.92, *p* = 0.235	*t* = −2.33, *p* = 0.040*	*t* = −1.92, *p* = 0.093
Prostaglandin E1		Drug dose	*t* = 1.78, *p* = 0.075	*t* = 4.18, *p* = 0.002**	*t* = 3.92, *p* = 0.002**
Atenolol		Weight	*t* = 1.21, *p* = 0.150	*t* = −5.48, *p* = 0.002**	*t* = −5.48, *p* = 0.002**
Heparin		Drug dose	*t* = 1.57, *p* = 0.136	*t* = 1.57, *p* = 0.136	*t* = 3.61, *p* = 0.003**
Enalapril		Drug dose	*t* = 0.89, *p* = 0.378	*t* = 1.99, *p* = 0.065	*t* = 3.96, *p* = 0.002**

* Statistically significant.

** Highly statistically significant.

## 4 Discussion

### 4.1 Principle Findings

In this study, specialists' perspectives on drug‐related side effects and associated risk factors in pediatric patients with CHD were investigated. The findings highlighted the most commonly reported side effects including hypotension, bradycardia, and gastrointestinal issues linked to medications such as Amiodarone, Dexmedetomidine, and Aspirin, respectively.

Amiodarone‐induced hypotension is of particular concern in CHD patients and may compromise cardiac output, as even minor blood pressure fluctuations may cause hemodynamic instability and increase the risk of organ hypo‐perfusion and progressive cardiac dysfunction [19]. Previous studies suggest that Amiodarone's complex pharmacokinetic profile, attributed to its high iodine content and prolonged elimination half‐life, necessitates rigorous side effect monitoring [20]. Furthermore, Amiodarone is associated with hypothyroidism—a clinically significant consideration in pediatrics due to the impact of thyroid dysfunction on growth and neurodevelopment [21].

Dexmedetomidine's strong sedative and sympatholytic effects can cause bradycardia, particularly in patients with arrhythmic tendencies, and require dose adjustment or discontinuation. As noted by Gertler et al., its alpha‐2 receptor agonism in the central nervous system reduces sympathetic outflow and decreases heart rate [22]. Additionally, its sedative properties pose significant risks of respiratory depression, especially in children with CHD who have underlying respiratory compromise [23, 24].

While Aspirin plays a crucial role in thrombosis prevention, its long‐term use may lead to gastrointestinal complications, including irritation and bleeding [25]. These Aspirin‐induced risk factors corroborate existing studies emphasizing pediatric patients' heightened susceptibility due to their thinner mucosal linings and prolonged drug exposure [25, 26, 27]. For pediatric patients with CHD who are on Aspirin therapy, prophylactic gastroprotective agents or alternative antiplatelet medications such as clopidogrel may be considered for those developing gastrointestinal symptoms [26].

Atenolol was associated with several risk factors in this study—including palpitations, vertigo, cold extremities, and bronchospasm—that may compromise treatment adherence. This is particularly concerning in pediatric patients unable to express discomfort or experience distress from symptoms like dizziness and cold extremities [13]. The risk of bronchospasm aligns with literature highlighting beta‐blockers' potential to provoke asthmatic symptoms, particularly in children with compromised pulmonary function or a history of asthma [27]. Given these side effects, clinicians should adopt a cautious prescribing approach, typically initiating therapy with gradual dose titration to mitigate side effects [9].

Furosemide‐related hypokalemia emerged as a notable concern, aligns with prior findings, necessitating potassium supplementation and regular electrolyte monitoring in pediatric use [28]. Hypovolemia is similarly problematic in children with heart failure, where stable perfusion is vital. Combining Furosemide with potassium‐sparing diuretics like Spironolactone may reduce these risks, though careful oversight remains essential [29].

Heparin use in pediatric CHD patients was considered to be associated with thrombocytopenia and postoperative bleeding, consistent with prior evidence of platelet reduction and major hemorrhage risks, especially following recent surgeries or invasive procedures [30].

Ibuprofen was noted for its potential to increase necrotizing enterocolitis risk in neonates/infants, consistent with previous pediatric studies [31, 32] and carried respiratory and intraventricular hemorrhage risks [32]. For pediatric pain/anti‐inflammatory needs, acetaminophen—though less potent—may be preferable [33].

Prostaglandin E1 was associated with a unique set of side effects, including apnea, hypoventilation, and fever, consistent with existing evidence of its respiratory depression effects that may necessitate mechanical ventilation [34, 35]. Apnea is particularly critical in infants with duct‐dependent lesions, who are at risk of compromised oxygenation [36].

Based on the results, dosage, polypharmacy, and age significantly influenced risk factor prevalence and severity. Precise dosing proved critical for narrow therapeutic index drugs like Digoxin where minor overdoses can lead to digoxin toxicity, arrhythmias, and dangerous bradycardia [37, 38]. Pediatric patients, particularly infants with immature renal systems, are at heightened risk due to their body's low capacity for drug clearance [11], necessitating regular monitoring of serum digoxin levels and careful dose adjustments [39]. For instance, Ibuprofen poses risks of renal toxicity and necrotizing enterocolitis in neonates due to their reduced renal clearance and heightened gastrointestinal vulnerability [40].

Polypharmacy was also associated with an increased risk of adverse effects. For instance, concurrent use of anticoagulants such as Heparin and Aspirin heightened bleeding risk [41], while combining Amiodarone with beta‐blockers or calcium channel blockers increased the likelihood of bradycardia and hypotension through cumulative effects on heart rate and vascular tone [42]. These risks are magnified in pediatric CHD patients because of underdeveloped metabolic pathways and compromised organ function, emphasizing the need for comprehensive medication review and individualized therapy [43, 44]. Moreover, underlying conditions such as heart failure and arrhythmias can alter pediatric responses to medications like Furosemide and Flecainide, exacerbating side effects due to unique pharmacodynamic challenges [13].

Differences in perceived drug‐related side effects and risk factors across clinician demographics mirrored findings from previous studies, highlighting the influence of personal and professional backgrounds on clinical decision‐making and risk assessment. Montastruc et al. noted higher adverse event reporting by female clinicians for cardiovascular medications [45], while Savarino et al. found older healthcare professionals were more cautious with prescribing nonsteroidal anti‐inflammatory drugs due to gastrointestinal concerns, showing the increased recognition of Indomethacin‐related necrotizing enterocolitis [46]. Additionally, studies revealed that experienced cardiologists and neonatologists, respectively, are more skilled at identifying drug‐specific complications, including Enalapril‐induced hyperkalemia in patients with renal impairment [47] and electrolyte imbalances associated with Prostaglandin E1 therapy in preterm infants requiring ductal patency [36]. These findings highlight the importance of tailored education and interdisciplinary collaboration to bridge perception gaps and standard risk assessment across varying demographic backgrounds.

In light of these findings, the medications assessed in this study can be broadly categorized into three therapeutic groups: (1) cardiovascular and antiarrhythmic agents such as Amiodarone, Digoxin, and Atenolol; (2) anti‐inflammatory and sedative agents including Ibuprofen and Dexmedetomidine; and (3) antithrombotic and diuretic therapies such as Heparin, Aspirin, Furosemide, and Prostaglandin E1. The side effects observed across these groups can be similarly classified into major clinical domains: cardiovascular complications (e.g., hypotension, arrhythmias, bleeding), respiratory effects (e.g., apnea, hypoventilation, bronchospasm), gastrointestinal symptoms (e.g., irritation, bleeding, necrotizing enterocolitis), and metabolic/electrolyte disturbances (e.g., hypokalemia, thyroid dysfunction).

These findings are consistent with prior studies, including Abdelghani et al., who reported an elevated bleeding risk in neonates receiving anticoagulants due to immature coagulation systems [48], and Smith et al., who noted a high incidence of postoperative tachyarrhythmias with milrinone, particularly in patients undergoing cardiopulmonary bypass [49]. Moreover, the impact of age and developmental stage—as emphasized by Mital —was reinforced in this study, with infants demonstrating increased vulnerability to drug toxicity due to immature enzymatic and receptor profiles [50]. Similarly, non‐cardiovascular complications like pressure injuries associated with corticosteroids and anticoagulants have been noted to occur more frequently in patients with congenital heart disease [51]. Collectively, these insights underscore the necessity of age‐adjusted dosing, vigilant monitoring, and judicious use of polypharmacy in pediatric CHD care to mitigate cumulative risks.

### 4.2 Implications for Practice

The findings underscore the necessity for personalized pharmacotherapy and vigilant monitoring in pediatric CHD patients to effectively manage drug‐related side effects. Given the unique pharmacokinetic and pharmacodynamic considerations in this population, practitioners should employ risk‐based medication protocols, adjusting dosages and monitoring strategies to the specific health profiles of neonates, infants, and children.

High‐risk drugs (e.g., Furosemide and Digoxin) warrant regular electrolyte and cardiac function assessments to prevent toxicity. Clinical decision support systems could enhance safety by identifying drug interactions and providing patient‐specific medication adjustments. To further improve medication safety, targeted and continuous provider education on pediatric pharmacology and CHD‐specific side effects, and institutional policy implementation such as Electronic Health Records (EHR) alerts for personalized medication management, are recommended.

### 4.3 Study Limitations

This study had several limitations that may impact the generalizability and interpretation of the findings. First, the relatively small sample size—limited to pediatric cardiologists in a specific urban area—may not fully capture the broader diversity of practice patterns and experiences across different regions or healthcare settings. Additionally, reliance on self‐reported data introduces the potential recall and response biases, as clinicians' perceptions of side effects may vary based on their experience, familiarity with certain medications, or retrospective judgement. This could lead to over‐ or underreporting specific side effects, affecting the consistency and accuracy of the findings. Future studies should use real patient data, EHR data, or pharmacovigilance databases to mitigate these biases and improve drug safety assessments.

Another limitation might be related to the research design, which was a cross‐sectional study. Although this study helped to investigate specialists' opinions, demonstrating causal relationships between specific medications and side effects was not possible. Future longitudinal or prospective studies in which patients are tracked over time can provide stronger evidence of causality for better data analysis. The current findings may not fully capture the complex interactions between drug use, dosage, and side effects. In case of getting access to high‐quality data, applying advanced statistical techniques such as regression analysis or *χ* ^2^ tests could yield deeper insights into these relationships. Finally, further research incorporating real‐world pharmacokinetic monitoring data, direct patient‐reported outcomes, and interventional studies could offer a more comprehensive understanding of individualized pharmacotherapy in pediatric patients with CHD.

## 5 Conclusion

This study examined specialists' views on drug‐related side effects and risk factors in pediatric CHD patients. The findings highlighted the necessity of personalized treatment strategies and rigorous monitoring to minimize side effects and improve patient outcomes. By integrating these findings into clinical practice, healthcare providers can enhance medication safety for pediatric patients with CHD. Further multicenter research with diverse populations is essential to strengthen evidence‐based practice protocols for this vulnerable patient group.

## Author Contributions

**Esmaeel Toni:** conceptualization, formal analysis, writing – original draft, methodology, investigation, writing – review and editing. **Haleh Ayatollahi:** conceptualization, methodology, investigation, writing – review and editing, and supervision. **Reza Abbaszadeh:** validation and supervision. **Alireza Fotuhi Siahpirani:** validation.

## Ethics Statement

This study was performed in line with the principles of the Declaration of Helsinki. Ethics approval was granted by the Ethics Committee of Iran University of Medical Sciences (IR.IUMS.REC.1401.1007).

## Consent

A written informed consent was completed by all participants.

## Conflicts of Interest

The authors declare no conflicts of interest.

## Transparency Statement

The lead author Haleh Ayatollahi affirms that this manuscript is an honest, accurate, and transparent account of the study being reported; that no important aspects of the study have been omitted; and that any discrepancies from the study as planned (and, if relevant, registered) have been explained.

## Data Availability

The data that support the findings of this study are available from the corresponding author upon reasonable request.
